# Chronic Testosterone Replacement Exerts Cardioprotection against Cardiac Ischemia-Reperfusion Injury by Attenuating Mitochondrial Dysfunction in Testosterone-Deprived Rats

**DOI:** 10.1371/journal.pone.0122503

**Published:** 2015-03-30

**Authors:** Wanpitak Pongkan, Siriporn C. Chattipakorn, Nipon Chattipakorn

**Affiliations:** 1 Cardiac Electrophysiology Research and Training Center, Faculty of Medicine, Chiang Mai University, Chiang Mai, Thailand; 2 Cardiac Electrophysiology unit, Department of Physiology, Faculty of Medicine, Chiang Mai University, Chiang Mai, Thailand; 3 Department of Oral Biology and Diagnostic Sciences, Faculty of Dentistry, Chiang Mai University, Chiang Mai, Thailand; 4 Center of Excellence in Cardiac Electrophysiology Research, Chiang Mai University, Chiang Mai, Thailand; Emory University, UNITED STATES

## Abstract

**Background:**

Although testosterone deficiency is associated with increased risks of heart disease, the benefits of testosterone therapy are controversial. Moreover, current understanding on the cardiac effect of testosterone during cardiac ischemia-reperfusion (I/R) periods is unclear. We tested the hypothesis that testosterone replacement attenuates the impairment of left ventricular (LV) function and heart rate variability (HRV), and reduces the infarct size and arrhythmias caused by I/R injury in orchiectomized (ORX) rats.

**Methodology:**

ORX or sham-operated male Wistar rats (n = 24) were randomly divided and received either testosterone (2 mg/kg, subcutaneously administered) or the vehicle for 8 weeks. The ejection fraction (EF) and HRV were determined at baseline and the 4^th^ and 8^th^ week. I/R was performed by left anterior descending coronary artery ligation for 30 minutes, followed by a 120-minute reperfusion. LV pressure, arrhythmia scores, infarct size and cardiac mitochondrial function were determined.

**Results:**

Prior to I/R, EF and HRV were impaired in the ORX group, but were restored in the testosterone-treated group. During I/R, arrhythmia scores and the infarct size were greater, and cardiac mitochondrial function was impaired, whereas the time to 1^st^ VT/VF onset and the LV end-systolic pressure were decreased in the ORX group when compared to the sham group. Testosterone replacement attenuated the impairment of these parameters in ORX rats during I/R injury, but did not show any benefit or adverse effect in non-ORX rats.

**Conclusions:**

Testosterone replacement restores cardiac function and autonomic regulation, and exerts cardioprotective effects during the I/R period via mitochondrial protection in ORX rats.

## Introduction

Androgen deficiency commonly occurs in middle aged to older men and sometimes includes younger men with underlying hypothalamopituitary or testicular disorders [1]. It is also known that testosterone deficiency is a risk factor for cardiovascular diseases (CVD) and coronary artery diseases (CAD) [2–4]. Previous studies have shown that there is a correlation between men with testosterone deficiency or low serum testosterone and an increased prevalence and risk of CVD and CAD [4–6], thus suggesting there may be a possible role for testosterone replacement therapy as a potential therapeutic strategy to prevent CVD in those groups of patients [7–9].

Although physiological testosterone replacement is an inexpensive and well-tolerated therapy, it does require careful monitoring [10]. Recent studies suggested a beneficial effect of physiological testosterone replacement therapy on lipid profiles [11] and ischemic insults of the heart [12, 13]. In hearts subjected to ischemia-reperfusion (I/R) injury, previous *in vitro* and *ex vivo* studies reported that testosterone exerts a cardioprotective effect through androgen receptors [1, 12–16]. Despite these reports, the roles of testosterone replacement therapy are still controversial since inconsistent reports exist regarding the effects of testosterone on the heart which had undergone I/R injury [17, 18]. Furthermore, information regarding the roles of testosterone deficiency/replacement in the I/R hearts were obtained from studies using *ex vivo* models [12, 19–21], making it difficult to translate these findings into the clinical setting.

In the present study, the effect of testosterone replacement was investigated on the left ventricular function, cardiac autonomic regulation being determined by the heart rate variability (HRV), cardiac arrhythmias, myocardial infarct size, cardiac mitochondrial function and the underlying mechanism in the I/R hearts of testosterone-deprived rats using an *in vivo* study model. We tested the hypothesis that testosterone-deprived rats develop left ventricular (LV) dysfunction and depressed HRV, and also that testosterone replacement attenuates the impairment of LV function and HRV, reduces cardiac arrhythmias and myocardial infarction size and attenuates cardiac mitochondrial dysfunction and apoptosis caused by I/R injury.

## Materials and Methods

### Animal preparation

All experiments were approved by the Institutional Animal Care and Use Committee of the Faculty of Medicine, Chiang Mai University, Chiang Mai, Thailand. Adult male Wistar rats weighing 300–350 grams were obtained from the National Laboratory Animal Center, Mahidol University, Bangkok, Thailand. All animals were housed in a climate controlled room (22–25°C with a 12-hour light/dark cycle) and fed on a normal diet of standard pelletized rat food and water *ad libitum*.

### Orchiectomy procedure

Rats were anesthetized and maintained using Isoflurane 2–3% in a dorsal recumbent position, and the skin of the scrotal area was shaved and scrubbed by sterile technique. An orchiectomy was performed using the scrotal approach technique [22]. A small incision was made at the tip of the scrotum, and the tunica vaginalis was incised and the testis, vas deferens and the spermatic blood vessels were exposed. Then, the blood vessels and vas deferens were ligated using absorbable sutures. The testis and epididymal fat pad was removed using the open technique. When the bleeding stopped, the remaining tissues were returned into the sac and the procedure repeated on the opposite testis. The skin incision was closed with a non-absorbable suture. Rats were monitored carefully to prevent any chewing of sutures and other complications. An analgesic drug and antibiotics were injected subcutaneously for 3 days after the operation.

### Surgical procedure of myocardial I/R

Rats were anesthetized by intramuscular injection of Zolitil (ZolazepamTiletamine) 50 mg/kg combined with Xylazine 3 mg/kg [23]. Then, tracheotomies were performed via a ventral midline incision. Rats were ventilated via the tracheotomy with room air from a positive pressure ventilator (Harvard Apparatus, Massachusetts, USA) to maintain PCO_2_, PO_2_, and pH parameters under physiological conditions. A Lead II electrocardiogram (ECG) (PowerLab 4/25 T, AD Instrument) was monitored throughout the study. The right carotid artery was canulated with a pressure conductance catheter (Scisense Instrument, Ontario, Canada) to measure the left ventricular pressure during the I/R period. A left-side thoracotomy at the fourth intercostal space was performed and the heart was exposed. The heart was allowed to stabilize for 15 minutes and then the left anterior descending coronary artery (LAD) was ligated at approximately 2 mm from its origin by a 5–0 silk suture with an atraumatic surgical needle threaded through a small stainless steel snare for the coronary artery occlusion [23]. Ischemia was confirmed by an ST elevation on the ECG and regional pallor of myocardial tissues of the ischemic area. I/R injury was induced by 30 minutes of ischemia, followed by 120 minutes of reperfusion.

### Experimental protocol

Rats were randomly divided into 2 groups (n = 16/group); a sham operated group and an orchiectomized (ORX) group. One week after orchiectomy, rats in each group were divided into two subgroups (n = 8/group) to be treated with either castor oil (control) or testosterone (2 mg/kg, subcutaneous injection) daily for 8 weeks. HRV and echocardiograms were carried out 3 times throughout the entire experiment; before the orchiectomy, then 4 and 8 weeks after the testosterone treatment. At the end of testosterone treatment (week 8), all rats underwent cardiac I/R, and cardiac function and arrhythmia parameters were determined in all groups. At the end of the experiment, the heart was removed to determine myocardial infarction size, cardiac mitochondrial function and a western blot analysis was carried out for assessment of apoptosis and gap junction protein.

### Heart rate variability (HRV) determination

Prior to the I/R study, the HRV was used to investigate cardiac autonomic regulation. The electrocardiogram (ECG) was recorded for 20 minutes in each rat using the PowerLab system (PowerLab 4/25T, AD instrument) with chart 5.0. Data from the ECG recording was analyzed using the MATLAB program [24]. Power spectra of RR interval variability were obtained by the Fast Fourier Transform (FFT) algorithm and the three major oscillatory components including high frequency (HF), low frequency (LF) and very low frequency (VLF) were determined. Parasympathetic activity was regarded as HF (0.6–0.3 Hz). Association of sympathetic and parasympathetic activity was regarded as LF (0.2–0.6 Hz). To minimize the effect of changes in total power on the LF and HF components, LF and HF were expressed as normalized units (LFnu and HFnu) by dividing LF or HF by the total power minus VLF. The LF/HF ratio was considered as an index of cardiac sympathovagal tone balance [25, 26]. An increased LF/HF ratio indicates a cardiac sympathovagal imbalance [27].

### Cardiac function determination

Prior to the I/R protocol, left ventricular function was assessed using an echocardiograph (SONOS4500, Philips). The chest area was shaved and rats were stabilized in a supine position. The probe was gently placed on the chest and moved to enable the collecting of data along the short and long axes of the heart. Signals from M-mode echocardiography at the level of the papillary muscles were recorded. Parameters obtained from the echocardiogram including left ventricular internal dimensions during systole (LVIDs) and diastole (LVIDd), and left ventricular posterior wall thickness during systole (LVPWs) and diastole (LVPWd) were recorded. Fractional shortening (FS) was calculated using the formula: %FS = (LVIDd—LVIDs) x 100 / LVIDd.

At the end of the experiment, left ventricular pressure during I/R injury was evaluated using an intravascular pressure catheter. The right carotid artery was canulated and the pressure in the LV chamber was recorded using a LabScribe2 program (iWorx Systems Inc., Dover, NH, USA) [23, 28]. Heart rate (HR), left ventricular end-systolic pressure (LVESP) and left ventricular end-diastolic pressure (LVEDP) were assessed prior to the occlusion of the LAD, at the end of ischemic period and at the end of reperfusion period.

### Arrhythmia determination during the I/R period

The ECG (Lead II) was monitored throughout the I/R period using PowerLab 4/25 (AD Instruments, Inc., Colorado Springs, CO, USA). The occurrence of arrhythmia was characterized using the Lambeth Conventions [29] and arrhythmia scores were determined based on the following criteria [30]: *score 0* = premature ventricular contractions (PVCs) < 50 beats; *score 1* = PVC between 50 and 499 beats; *score 2* = PVC > 500 beats and/or one episode of spontaneously reverting ventricular tachycardia or ventricular fibrillation (VT/ VF); *score 3* = more than one episode of spontaneously reverting VT/VF (<1 min of total combined duration); *score 4* = 1–2 min of total combined VT/VF and *score 5* = >2 min of VT/VF. The VT/VF incidence and the time to first VT/VF onset were also determined.

### Infarct size determination

At the end of the experiment, the heart was removed from the body and the LAD was occluded again at the same site as previously during the ischemic period. The heart was mounted on the modified Langendorff apparatus via the aorta and irrigated with normal saline to wash out any blood from the chambers and vessels [23]. Evans blue (0.5%) was retrogradely infused through the aortic root. The area which was not infused and stained by Evan blue was defined as the area at risk (AAR). The heart was cut horizontally from the apex to the occluding site into 7–8 slices [23] and these were immersed and incubated in 37°C Triphenyltetrazolium chloride (TTC) for 12 minutes to distinguish the infarct tissues (white area) from the viable myocardium (red area). The area which demonstrated neither blue nor red was defined as the infarct site. The area measurements were performed with Image tool software version 3.0 [23].

### Cardiac mitochondrial function study

At the end of the experiment, cardiac mitochondria were isolated from non-ischemic and ischemic areas of the ventricles, and protein concentration was determined as previously described [23, 31]. Cardiac mitochondrial functions were determined by measuring the mitochondrial reactive oxygen species (ROS) production, mitochondrial membrane potential change (ΔΨm) and mitochondrial swelling [23, 28].

### Measurement of cardiac mitochondrial ROS production

Mitochondrial ROS production was determined by using Dichlorohydro-fluorescein diacetate dye (DCFDA) [32, 33]. The DCFDA diffused through the mitochondrial membrane and is deacetylated by intracellular esterases to a non-fluorescent compound which was later oxidized by ROS into dichlorofluorescein (DCF). DCF was a highly fluorescent compound which could be detected by fluorescence spectroscopy with maximum excitation. Increasing of DCF indicated the increasing of ROS production. Fluorescent intensity of the DCF was measured with an excitation wavelength at 485 nm and an emission wavelength at 530 nm by a fluorescent microplate reader and represented as arbitrary units (a.u.) of fluorescent intensity [23, 34].

### Measurement of cardiac mitochondrial membrane potential change (ΔΨm)

Mitochondrial membrane potential change was determined by using the dye 5,5′,6,6′-tetrachloro-1,1′,3,3′-tetraethylbenzimidazolcarbocyanine iodide (JC-1) and was determined as fluorescence intensity by using a fluorescent microplate reader [23, 34]. The JC-1 monomer (green fluorescence) and aggregate (red fluorescence) forms were excited at the same wavelength of 485 nm, whereas the emission of the JC-1 monomer and aggregate forms were detected at wavelength 590 nm and 530 nm, respectively. The change in mitochondrial membrane potential was calculated from the ratio of red to green fluorescence (red/green fluorescence intensity ratio). A decrease in the red/green fluorescence intensity ratio indicates mitochondrial membrane depolarization [23, 34].

### Measurement of cardiac mitochondrial swelling

The isolated mitochondrial suspension was used to determine mitochondrial swelling using a microplate reader (Synergy HT, Bio Tek, **Winooski**, Vermont, USA) to measure the change in the absorbance of the mitochondrial suspension at 540 nm (A540). Cardiac mitochondrial swelling was indicated by a decrease in the absorbance by the mitochondrial suspension [23, 34]. Transmission electron microscopy (TEM) was also used to determine the morphology of isolated cardiac mitochondria from both non-ischemic (remote) and ischemic areas [23].

### Western blot analysis

Heart tissues were collected from ischemic and non-ischemic (remote) areas, quickly frozen in liquid nitrogen and stored at −80°C until used [23]. Myocardial protein extract was prepared by a homogenization of nitrogen-frozen heart tissues in a lysis buffer (containing 1% Nonidet P-40, 0.5% sodium deoxycholate, 0.1% sodium dodecyl sulfate (SDS) in 1×PBS), and subsequently homogenized at 4°C, and then centrifuged at 13000 round/minute for 10 minutes and defined as total protein. The total protein was mixed with loading buffer [10% mercaptoethanol, 0.05% bromophenol blue, 75 mM Tris-HCl, pH 6.8, 2% sodium dodecyl sulphate (SDS) and 10% glycerol] and heated at 95°C for 10 min. Then, the protein samples were subjected to 10% or 15% SDS polyacrylamide gel electrophoresis (SDS-PAGE) and transferred to polyvinylidene difluoride membranes using a semi-dry transfer system (Trans-Blot SD; Bio-Rad, Hercules, CA, USA). The membranes were blocked with 5% non-fat skimmed milk in TBST (20 mM Tris-HCl, pH 7.6, 137 mM NaCl and 0.1% Tween-20) for 1 hour. The membranes were subsequently exposed to anti-Bax (Santa Cruz Biotechnology, Santa Cruz, CA, USA), anti-Bcl-2 (Cell Signaling Technology, Danvers, MA, USA), anti-total Connexin 43 (Santa Cruz Biotechnology, Santa Cruz, CA, USA), anti-Connexin 43 phosphorylated at S368 (Cell Signaling Technology, Danvers, MA, USA), or β-actin (Santa Cruz Biotechnology, Santa Cruz, CA, USA), overnight at 4°C. On the next day, the membranes were washed and incubated with horseradish peroxidase–conjugated secondary antibody (Cell Signaling Technology, Danvers, MA, USA) for 1 hour at room temperature. The signals were developed by incubating with an enhanced chemiluminescense reagent and subjected to autoradiography. The immunoblot films were scanned and the band density was analyzed using ImageJ analysis (NIH image).

### Statistical analysis

Data were expressed as mean ± SEM. Statistical comparison within groups was analyzed with a one-way ANOVA with a post-hoc LSD test, whereas statistical comparison between groups was analyzed using a Student’s *t*-test. A Chi-squared test was performed to compare the VT/VF incidence among groups. All statistical analysis was performed using SPSS version 10.0. The statistical significance was accepted at the level of a p-value less than 0.05.

## Results

### Testosterone replacement improved both cardiac function and HRV in ORX rats

**Fig 1:**
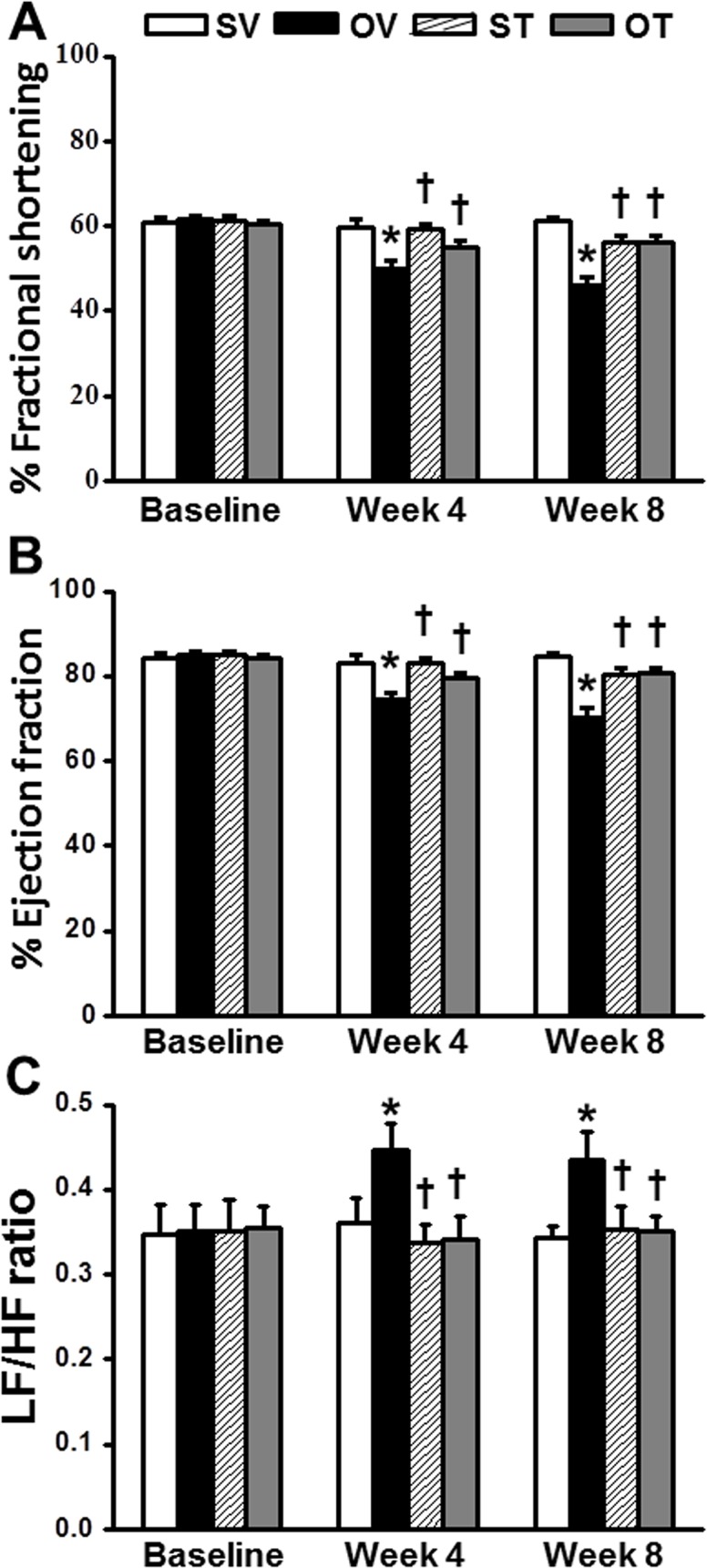
Effect of testosterone replacement on fractional shortening (FS), ejection fraction (EF) and heart rate variability (represent as Low frequency/High frequency ratio: LF/HF ratio) prior I/R injury. (A, B) Testosterone replacement restored the FS and EF at week 4 and 8, when compared with OV group. (C) Testosterone replacement reduced LF/HF ratio at week 4 and 8, when compared with OV group. *p < 0.05 vs. SV group, †p < 0.05 vs. OV group. SV = Sham+Vehicle, OV = ORX+Vehicle, ST = Sham+Testosterone, OT = ORX+Testosterone.

In ORX rats, serum testosterone was markedly decreased when compared with the sham rats (Table 1). Beginning at 4 weeks after ORX, the impairment of both cardiac function and cardiac autonomic balance (i.e. LF/HF ratio) was demonstrated. The echocardiogram showed that the fractional shortening (FS) and the ejection fraction (EF) were decreased significantly, whereas the LF/HF ratio was markedly increased, when compared with the sham group (Fig 1). Testosterone replacement significantly improved the cardiac function by increasing the FS (Fig 1(A)) and EF (Fig 1(B)), and improved cardiac sympathovagal balance by decreasing the LF/HF ratio (Fig 1(C)) in ORX rats.

**Table 1:** Summary of blood serum testosterone concentration at week 8.

Parameters	SV	OV	ST	OT
**Blood serum testosterone (ng/ml)**	3.4 ± 0.25	0.03 ± 0.01 *	4.3 ± 0.21 ^†^	3.6 ± 0.24 ^†^

SV = Sham+Vehicle, OV = ORX+Vehicle, ST = Sham+Testosterone, OT = ORX+Testosterone.

*p <0.05 vs. SV group

†p <0.05 vs. OV group.

### Testosterone improved left ventricular pressure during I/R injury in ORX rats

The changes in the left ventricular pressure at different time points are shown in Table 2. At baseline, the LVESP was significantly lower in ORX rats compared to the sham rats, whereas the heart rate and LVEDP showed no difference between the groups. However, testosterone replacement in ORX rats helped to restore the LVESP to the same level as in the sham rats (Table 2). During the I/R injury (i.e. at the end of ischemia and end of reperfusion), both ORX and sham rats that were given testosterone replacement had higher LVESP than the vehicle-treated ORX rats.

**Table 2:** Effects of Testosterone on heart rate and left ventricular pressure during I/R injury.

Parameter	Baseline				End of ischemia				End of reperfusion			
	SV	OV	ST	OT	SV	OV	ST	OT	SV	OV	ST	OT
**HR**	230.9±17	236.8±8	227.4±15	209.8±10	233.7±23.9	234.1±13.2	221.1±17.7	199.8±9.0	257.5±24.9	255.1±14.5	240.3±17.5	225.7±16.8
**LVESP**	113.1±6.4	94.6±4.1*	111.8±3^**†**^	113.4±3.5^**†**^	88.1±9.0	69.5±7.7	92.1±6.5^**†**^	92.4±10.2^**†**^	105.7±5.3	88.9±5.3	109.9±5.2^**†**^	113.4±5^**†**^
**LVEDP**	16.7±2.2	19.8±2.2	16.8±0.7	24.7±8.9	23.5±3.6	25.3±3.1	20±5.9	22.1±8.8	20.4±2.8	26.3±2.4*	21±8.1^**†**^	21.0±8.1^**†**^

SV = Sham+Vehicle, OV = ORX+Vehicle, ST = Sham+Testosterone, OT = ORX+Testosterone. HR = heart rate; LVESP = left ventricular end-systolic pressure; LVEDP = left ventricular end-diastolic pressure.

*p < 0.05 vs. SV group

†p < 0.05 vs. OV group.

### Testosterone decreased cardiac arrhythmias during I/R injury in ORX rats

**Fig 2:**
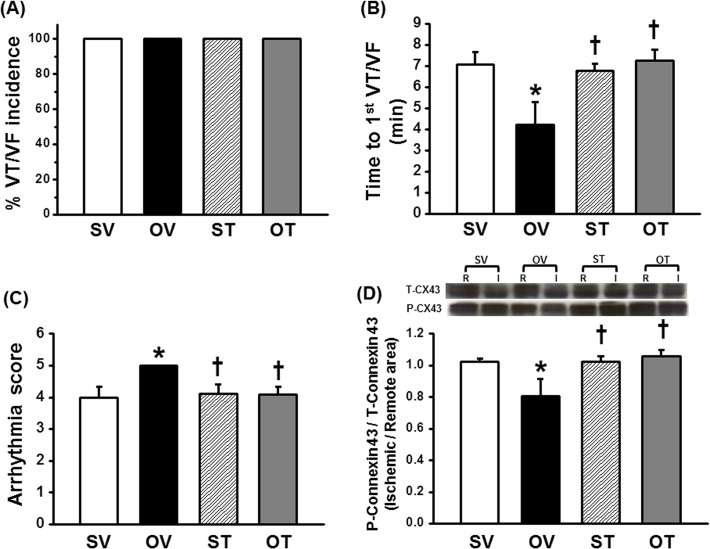
Testosterone and the occurrence of cardiac arrhythmia. Testosterone replacement did not reduce (A) VT/VF incidence but reduced cardiac arrhythmia by increasing (B) Time to 1^st^ VT/VF incidence and decreasing (C) arrhythmia score, compared to ORX group. (D) Testosterone increased P-Connexin 43/T-Connexin 43 ratio compared to OV group. *p < 0.05 vs. SV group, †p < 0.05 vs. OV group. SV = Sham+Vehicle, OV = ORX+Vehicle, ST = Sham+Testosterone, OT = ORX+Testosterone. VT = Ventricular tachycardia, VF = Ventricular fibrillation, R = Remote area, I = Ischemic area, CX43 = Connexin 43

During I/R, the VT/VF incidence showed no difference among all groups (Fig 2(A)). However, in ORX rats, the arrhythmia score was significantly greater, whereas the time to the first VT/VF onset was significantly lower, when compared with the sham group, indicating the high susceptibility to arrhythmias. Interestingly, testosterone replacement markedly attenuated cardiac arrhythmias during I/R by prolonging the time to the 1^st^ VT/VF onset (Fig 2(B)) and reducing the arrhythmia score (Fig 2(C)). Consistent with this finding, the western blot analysis of connexin 43 showed that the phosphorylated connexin 43 per total connexin 43 ratio (P-Connexin 43/T-Connexin 43 ratio) was significantly decreased in ORX rats treated with a vehicle, whereas testosterone replacement significantly increased P-Connexin 43/T-Connexin 43 ratios back to the level of that in the sham group (Fig 2D).

### Testosterone reduced the infarct size in I/R heart of ORX rats

**Fig 3:**
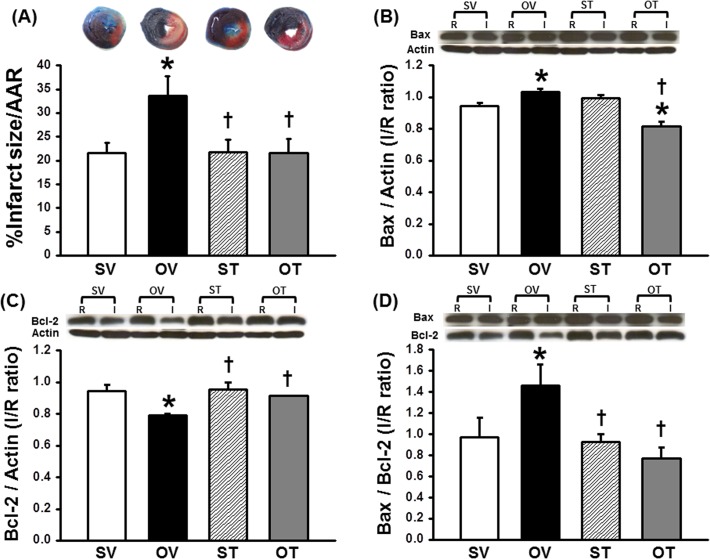
Effect of testosterone replacement on the infarct size and anti-apoptotic and pro-apoptotic proteins. The area at risk (AAR) showed no different among all groups. (A) Testosterone replacement reduced the infarct size by 36%, compared to the OV group. Testosterone attenuated apoptotic process as indicated by (B) increased anti-apoptotic Bcl-2, (C) decreased pro-apoptotic Bax expression, and (D) reduced Bax/Bcl-2 ratio, compared to the OV group. *p < 0.05 vs. SV group, †p < 0.05 vs. OV group. SV = Sham+Vehicle, OV = ORX+Vehicle, ST = Sham+Testosterone, OT = ORX+Testosterone, R = Remote area, I = Ischemic area

The area at risk (AAR) was not significantly different in all groups. However, the infarct size of ORX rats was significantly greater compared with the sham group (Fig 3A). ORX rats with testosterone replacement had smaller infarct size than in the vehicle-treated ORX rats, accounting for approximately 36% reduction in infarct size (Fig 3A).

Western blot analysis showed that anti-apoptotic protein Bcl-2 expression in the heart of ORX rats treated with vehicle was significantly decreased compared with the sham group, whereas the pro-apoptotic protein Bax expression was markedly increased (Fig 3B and 3C), compared with the sham group. Consistently, the Bax/Bcl-2 ratio in the ORX rats treated with vehicle was also increased (Fig 3D). ORX rats with testosterone replacement had increased Bcl-2 expression, decreased Bax expression and decreased Bax/Bcl-2 ratio, compared to the vehicle-treated ORX rats (Fig 3B–3D).

### Testosterone attenuated cardiac mitochondrial dysfunction in ORX rats with cardiac I/R injury

**Fig 4:**
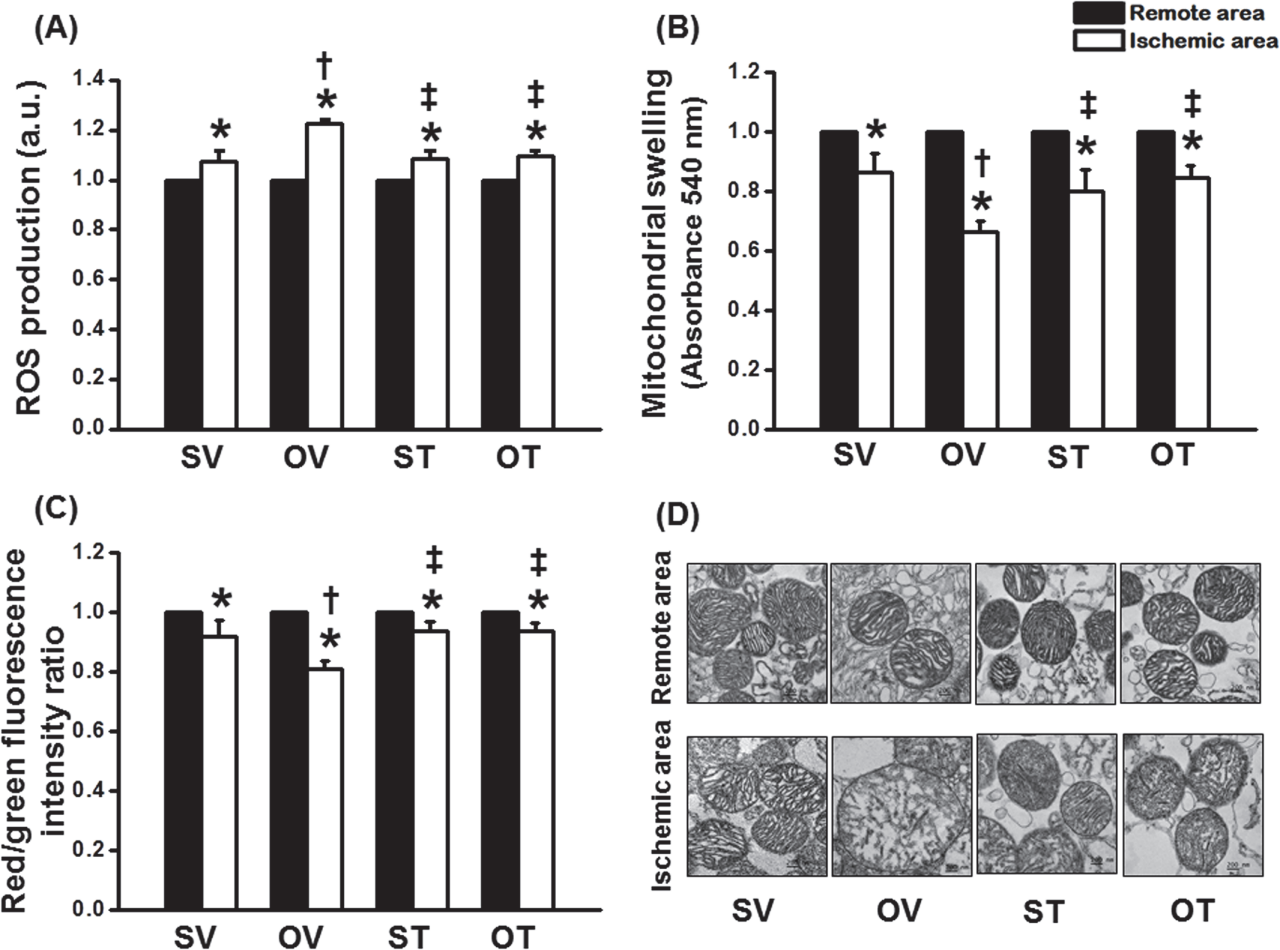
Effect of testosterone on cardiac mitochondria after ischemic-reperfusion period. Testosterone improved cardiac mitochondrial function by showing (A) reduced reactive oxygen species (ROS) production, (B) attenuated mitochondrial swelling and (C) attenuated mitochondrial membrane depolarization in ischemic area. (D) Testosterone also attenuated the deterioration of cardiac mitochondrial morphology. *p < 0.05 vs. Remote area, †p < 0.05 vs. SV group, ‡p < 0.05 vs. OV group. SV = Sham+Vehicle, OV = ORX+Vehicle, ST = Sham+Testosterone, OT = ORX+Testosterone

Cardiac mitochondria taken from the ischemic myocardium in ORX rats demonstrated increased mitochondrial ROS production (Fig 4A), increased mitochondrial swelling which was indicated by the decrease in absorbance (Fig 4B) and increased mitochondrial depolarization which was indicated by the decrease of the red/green fluorescent intensity ratio (Fig 4C), compared to the non-ischemic area. However, testosterone replacement in ORX rats attenuated cardiac mitochondrial dysfunction in the ischemic myocardium which was indicated by a reduction in mitochondrial ROS production (Fig 4A), decreased cardiac mitochondrial swelling (Fig 4B) and reduced cardiac mitochondrial depolarization (Fig 4C). Representative pictures of cardiac mitochondria taken from the transmission electron microscope (TEM) are shown in Fig 4D. Mitochondrial swelling, which is indicated by abnormal morphology together with the loss of cristae of the cardiac mitochondrion in the ischemic area, was observed in the ORX rats treated with the vehicle. In ORX rats with testosterone replacement, cardiac mitochondrial swelling was observed to be markedly reduced in the ischemic myocardium.

## Discussion

In this study, the cardioprotective effects of testosterone in testosterone-deprived rats heart with I/R injury were demonstrated. The major findings of this study are as follows. Prior to I/R injury, testosterone replacement provided cardioprotective effects in testosterone-deprived rats as indicated by (1) improved cardiac functions by markedly preserved %EF and %FS, and (2) attenuated cardiac sympathovagal imbalance by a markedly decreased LF/HF ratio. During the I/R period, testosterone replacement in ORX rats exerted the beneficial effects as indicated by (1) improved left ventricular pressure; (2) markedly decreased infarct size; (3) reduced fatal cardiac arrhythmias by increased time to 1^st^ VT/VF onset and reduced arrhythmia scores; and (4) attenuated cardiac mitochondrial dysfunction caused by I/R injury by reducing ROS production, cardiac mitochondrial swelling and mitochondria membrane depolarization.

In ORX rats, our results emphasized the importance of the physiological level of testosterone by demonstrating the adverse effects of testosterone deprivation on the left ventricular function and cardiac sympathovagal regulation. In this study, decreasing of FS and EF were observed starting at week 4 after orchiectomy, whereas testosterone replacement clearly demonstrated cardioprotective effects by improving the left ventricular function in the testosterone-treated group. This finding is consistent with the previous studies which also indicated that cardiac muscle is one of the target organs of testosterone hormone, which plays a beneficial role on cardiac function by improving cardiac contractility and improved calcium regulation [35, 36]. In addition to impaired left ventricular function in ORX rats, testosterone deprivation also significantly affected the cardiac autonomic tone balance as shown by an increased LF/HF ratio (i.e. so-called depressed HRV) in ORX rats. We found that depressed HRV was initially observed in week 4 after ORX, whereas testosterone replacement could restore the HRV in the testosterone-treated group. This result is consistent with a previous clinical report in men with stable coronary artery disease (CAD) which demonstrated that a high level of blood testosterone was associated with reduced sympathovagal imbalance [37]. Since depressed HRV is known to be associated with increased oxidative stress [38, 39] and that testosterone deprivation has been shown to affect the antioxidant defense system in the left ventricle (LV) [40] and associated with the increased oxidative stress [41, 42], testosterone replacement could play a crucial role in the protection of cardiac sympathovagal imbalance by reducing the oxidative stress and the enhancing of the antioxidant defense system. This hypothesis is supported by the findings of this study that ORX rats had increased cardiac mitochondrial ROS production, and testosterone attenuated ROS level.

During the I/R period, the results clearly demonstrated that ORX rats treated with testosterone had a higher LVESP than in the untreated group, indicating that testosterone plays a beneficial role in the post-ischemic functional recovery. This finding is consistent with previous reports using ORX rats with I/R and myocardial infarction models which demonstrated that chronic testosterone replacement (physiological and supraphysiological dose) confers cardioprotection by maintaining intracellular calcium homeostasis [21, 43]. However, inconsistent reports exist which showed that acute administration of testosterone at a physiological level could depress the recovery of myocardial function during I/R injury by inducing hypertrophic response in the heart via androgen receptors, resulting in an increase of ventricular stiffness [19]. These discrepancies in findings regarding the role of testosterone on the cardiac function during I/R could be due to differences in the experimental model. Nevertheless, the findings of this study demonstrated for the first time in *in vivo* that chronic administration of testosterone improved left ventricular function during I/R.

During I/R injury, this study clearly demonstrated that ORX rats were susceptible to arrhythmias as indicated by a shorter interval of time to 1^st^ VT/VF onset and higher arrhythmia scores than those in the control group, while testosterone replacement in ORX rats had a longer time to 1^st^ VT/VF onset and lower arrhythmia scores. This finding is consistent with a previous study in rats which demonstrated that the physiological dose of testosterone (2 mg/kg) combined with adrenergic stimulation could reduce reperfusion arrhythmias during I/R injury by reducing the incidence of a premature ventricular beat [12]. It is possible that the mechanism that testosterone attenuated cardiac arrhythmias during I/R injury was involved with connexin 43 phosphorylation. It has been shown that the phosphorylation of connexin 43 at serine 368 residue plays an essential role in preserving cell to cell communication through gap junctions in the myocardium, and that decreased connexin 43 phosphorylation could facilitate arrhythmias [23, 31, 44]. This study demonstrated that testosterone-deprived rats had decreased connexin 43 phosphorylation, and that testosterone treatment increased the phosphorylation of connexin 43, resulting in increased cell to cell communication, and fatal arrhythmias were attenuated during the I/R period.

The myocardial infarct size has been shown to be associated with the severity of left ventricular dysfunction as well as mortality rate [23, 31, 45, 46]. Since myocardial infarction plays an essential role in cardiac dysfunction, reduction in the infarct size would be of great benefit regarding contractility. In this study, it was demonstrated that testosterone replacement in ORX rats reduced the infarct size caused by I/R injury by 36% when compared to the untreated group. This finding is consistent with previous studies that testosterone could significantly reduce the infarct size in the hearts subjected to I/R injury [12, 43]. The mechanism responsible for infarct size reduction in the testosterone-treated ORX rats could be due to the reduced apoptosis and the reduction of cardiac mitochondrial dysfunction. In this study, it was found that testosterone attenuated myocardial apoptosis by increasing anti-apoptotic (Bcl-2) proteins and reducing pro-apoptotic (Bax) proteins.

Moreover, cardiac mitochondrial dysfunction, as indicated by increased mitochondrial ROS production, mitochondrial depolarization and mitochondrial swelling, was prominent in the ischemic myocardium of ORX rats subjected to I/R injury, and these dysfunctions were attenuated by the testosterone replacement. Mitochondria are known to play an essential role in the cell survival especially in cardiomyocytes [47]. During an I/R period, the oxidative phosphorylation rate at the inner mitochondrial membrane is reduced, leading to a decrease in the energy production and causing a rapid increase in ROS production [48]. When the accumulation of ROS is high enough to reach a critical threshold level [49], it triggers the opening of the mitochondrial permeability transition pores (mPTP) or the inner membrane anion channels (IMAC), resulting in the collapse of the mitochondrial membrane potential which is known as mitochondrial membrane depolarization [49–51]. Moreover, the prolonged opening of mPTP could lead to bi-directional diffusion of low molecular weight molecules across the inner mitochondrial membrane. Since high molecular weight molecules (i.e. proteins) remain in the matrix, this leads to an increase in the matrix osmotic pressure and resulting in mitochondrial swelling and/or mitochondrial membrane rupture. This will cause the release of cytochrome c and other proapoptotic proteins, leading to apoptotic cell death [52, 53]. Since testosterone has been shown to reduce ROS generation [42] and suppress oxidative stress [54], it is possible that this anti-oxidative effect of testosterone could be responsible for its cardioprotection in this study. This hypothesis is supported by our findings that testosterone attenuates cardiac mitochondrial ROS levels and reduced mitochondrial dysfunction during I/R injury. These beneficial effects of testosterone replacement in ORX rats could also play an important role in decreasing the cellular apoptotic process, infarct size and cardiac arrhythmias as well as improving LV function during I/R injury.

## Conclusions

Testosterone replacement exerts cardioprotective effects by improving left ventricular function and cardiac sympathovagal balance impaired by testosterone deprivation in ORX rats. Chronic testosterone replacement also ameliorates left ventricular dysfunction, and reduces the infarct size and cardiac arrhythmias impaired by I/R injury under testosterone-deprived conditions. The mechanisms responsible for these beneficial effects of testosterone could be due to its ability to attenuate cardiac mitochondrial dysfunction and cardiomyocyte apoptosis. These findings provide important information regarding the cardioprotective benefits of testosterone in testosterone-deprived subjects with and without I/R injury. Future clinical studies are needed to determine its clinical usefulness in patients with acute myocardial infarction.
